# Health, Wealth, and Well-Being

**Published:** 2007-09-15

**Authors:** Lynne S. Wilcox

**Figure:**
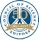
This article is part of PCD’s participation in the Council of Science Editors’ Global Theme Issue on Poverty and Human Development. Other PCD articles in this series are: *Unnatural Causes: Is Inequality Making Us Sick?*

Today, October 22, 2007, *Preventing Chronic Disease* (*PCD*) joins *Morbidity and Mortality Weekly Report*, *Emerging Infectious Diseases,* and scientific journals around the world in publishing on the topic of poverty and human development. This joint effort is sponsored by the Council of Science Editors (1), and the global partnership of more than 200 journals represents both developed and developing countries. *PCD* is honoring this commitment with a special addition to our October 2007 issue on society and health.

The association of poverty and poor health has been recognized for centuries. However, as Waitzkin has observed, researchers currently studying the effects of social determinants on health rarely credit previous generations for making similar observations and reaching similar conclusions (2). Waitzkin's essay explores the work of 19th-century Prussian pathologist and public health visionary Rudolf Virchow. One of the most remarkable aspects of Virchow's career was his ability to span the divide between cellular pathology (the essence of medical bench science) and public health (the discipline of population wellness). Medical students are still taught principles elucidated in his comprehensive text, *Cellular Pathology*, but Virchow also studied health differences across social classes. "The improvement of medicine," Virchow said, "would eventually prolong human life, but improvement of social conditions could achieve this result even more rapidly and successfully" (3).

In this issue of *PCD*, Braveman discusses highlights of the most recent investigations into the poverty–health link; these investigations have led to consensus that the link is causal (4). That poverty leads to poor health is an established fact, but what are the associated questions for practice and policy? Members of the public health community may ask, "How shall we address this problem?" but a significant portion of the U.S. public asks, "Does society have a responsibility to address it?" Put another way, it is not a universally held belief in the United States that all citizens have inherent rights to good health. This aspect of the political will to support health rights is not subject to scientific argument; it represents the values of a society.

The California Endowment recently commissioned several studies that examined the beliefs of California citizens about health and society. The first of these studies was described in *Health Individualism: Findings From Cognitive Elicitations Among Californians* (5) and was derived from interviews with men and women of all ages and income levels, representing diverse cultures and political beliefs. Regardless of their backgrounds, interviewees indicated that they believed good health was the result of individual behaviors and that the consequences of those behaviors rested solely on the individual. These respondents did not independently consider whether individual health might depend on circumstances outside the individual's control or whether healthy citizens could improve the overall function of society. When interviewers introduced the concepts of healthy communities or disparities in health, the respondents did not accept them as major principles. In fact, the subjects had difficulty conceptualizing public health in any form.

The second study, described in the paper *Civic Wellbeing: An Analysis of Qualitative Research Among California Residents* (6), examined how to focus public awareness on environments that affect health. The report's data were drawn from focus groups, members of which represented a diverse portion of the California population. Three models of health in society were presented to the subjects: 1) better policy choices prevent health problems, 2) policy decisions have an impact on public environments and public health, and 3) healthy environments lead to a community's economic well-being. Researchers found that the most appealing model was the second one, also called the *public environment frame*.

The second study used hypothetical newspaper articles to assess the subjects' reactions to the three models. The key themes of the public environment frame were health–environment connections and collective action to improve the community. This framing readily led respondents to accept the priority needs of poor communities without blaming these communities' members. Perhaps most intriguing, "civic well-being" and "public environment" were terms that resonated with the focus group participants, in contrast to the "healthy communities" and "health disparities" concepts rejected by the participants from the health individualism study.

These findings are limited to small, though well-designed, qualitative studies in California. But the findings suggest that reciting statistics on the connection between economic disparities and health will not be effective in motivating political will. Instead, discussing real opportunities to improve civic well-being, support good citizenship, and promote effective government encourages people to include health in their vision of community. The World Health Organization defines health as "a complete state of physical, mental and social well-being, and not merely the absence of disease or infirmity" (7). However, for most people, well-being, rather than health, is the larger concept. Perhaps translating this understanding from the individual to the community level can be an effective way of developing a broader base of political will for social action. Creating opportunities for citizens across the socioeconomic spectrum to strengthen community and governance, rather than asserting society's responsibility for each individual's health, may provide the common value. It is fair to ask whether the most disadvantaged populations would be well served by such an approach. But Marmot's extensive research on social standing and health offers reassurance that people in all classes can ameliorate health disparities though social participation (8).

The selection of articles in *PCD*'s July 2007 issue on community wellness and October 2007 issue on society and health includes several discussions of potential community solutions. For this addition to our October issue, we have included a film clip from the upcoming Public Broadcasting Service series on "*Unnatural Causes: Is Inequality Making Us Sick?*" This seven-part series explores the association between socioeconomic status and health in selected American communities (9). One of the most striking aspects of this story is that, although the United States spends more money per capita on health care than any other country in the world, it ranks 30th in life expectancy. Furthermore, the risk of early death in low social classes is greater compared with risk in high social classes, and this gap has been steadily increasing over the last 30 years.

In 1848, Virchow published a report on the typhus epidemic in Upper Silesia, a poverty-stricken region of Germany (10). He mentioned the treatment of individuals, described the government's response, and then devoted most of the report to recommendations for "safeguarding the future." Virchow commented that the population was poor and starving before the epidemic occurred. "There can no longer be any doubt that such an epidemic dissemination of typhus could only have been possible under the wretched conditions of life that poverty and lack of culture had created in Upper Silesia" (10). To prevent this catastrophe in the future, Virchow wrote that it would be necessary to promote education, transportation, agriculture, and manufacturing. That is, improving the economy and reducing poverty in the region would improve the inhabitants' health.

These social and economic elements function at the community level. Encouraging citizens to influence these powerful social engines may create political will that can counter the effects of poverty and improve the health of all citizens. As Virchow observed, "Medicine is a social science, and politics is nothing more than medicine in larger scale" (3).
